# Supplementary material for: Grapevine Xylem Sap Is a Potent Elicitor of Antibiotic Production in Streptomyces spp
Source: Antibiotics (Basel). 2022 May 17;11(5):672. doi: 10.3390/antibiotics11050672 (PMC9137808; doi:10.3390/antibiotics11050672)
Supplement: Supplementary file 1 [file antibiotics-11-00672-s001.zip › antibiotics-1722047-supplementary.pdf]

Supplementary tables and figures

Table S1: Experimental monoisotopic masses and its correspondent theoretical values from detected adducts or characteristic fragments of putatively identified compounds in peaks elicited by grapevine xylem sap.

Strain	Medium	Dereplicated component	Molecular formula	Adduct	Calculated m/z	Experimental m/z	Error (ppm)
S. coelicolor M145	NMMP+SAP	Deoxydehydrochorismic acid	$C_{10}H_8O_5$	$M+Na^+$	231,0264	231,0261	-1,3
S. coelicolor M145	NMMP+SAP	ε-Actinorhodin	$C_{32}H_{24}O_{15}$	$M+H^+$	649,1188	649,1196	1,2
S. coelicolor M145	NMMP+SAP	γ-Actinorhodin	$C_{32}H_{22}O_{14}$	$M+NH4^+$	648,1348	648,1349	0,2
S. coelicolor M145	NMMP+SAP	-	$C_{15}H_{15}NO_6$	$M+H^+$	306,0972	306,0972	0,0
S. coelicolor M145	NMMP+SAP	-	$C_{32}H_{24}O_{14}$	$M+NH4^+$	650,1504	650,1505	0,2
S. coelicolor M145	YEPD+SAP	B 26 / Anhydro SEK-4B	$C_{16}H_{12}O_6$	$M+H^+$	301,0707	301,0705	-0,7
S. coelicolor M145	YEPD+SAP	SEK-34	$C_{16}H_{14}O_6$	$M+H^+$	303,0863	303,0860	-1,0
S. coelicolor M145	YEPD+SAP	SEK-34B	$C_{16}H_{12}O_5$	$M+H^+$	285,0758	285,0757	-0,4
S. coelicolor M145	YEPD+SAP	γ-Actinorhodin	$C_{32}H_{22}O_{14}$	$M+NH4^+$	648,1348	648,1350	0,3
S. ambofaciens	YEPD+SAP	Nocardamine	$C_{27}H_{48}N_6O_9$	$M+H^+$	601,3556	601,3555	-0,2
S. griseus subsp. *griseus*	YEPD+SAP	Chromomycin A3	$C_{57}H_{82}O_{26}$	Fragment $C_{36}H_{49}O_{16}^+$	737,3015	737,3003	-1,6
				Fragment $C_{42}H_{59}O_{19}^+$	867,3645	867,3636	-1,0
S. argillaceus	YEPD+SAP	1H-Indole-5-carboxylic acid	$C_9H_7NO_2$	$M+H^+$	162,0550	162,0549	-0,6
S. argillaceus	YEPD+SAP	Xanthicin	$C_{13}H_{15}NO_5$	$M+H^+$	266,1023	266,1018	-1,9
S. argillaceus	YEPD+SAP	-	$C_{13}H_8N_2O_3S$	$M+H^+$	273,0329	273,0323	-2,2
S. argillaceus	YEPD+SAP	Mithramycin A	$C_{52}H_{76}O_{24}$	$M+NH4^+$	1102,5065	1102,5061	-0,4
S. olivaceus	YEPD+SAP	Antibiotic BA 12100MY1	$C_{25}H_{24}O_9$	$M+H^+$	469,1493	469,1492	-0,2
S. olivaceus	YEPD+SAP	-	$C_{28}H_{29}NO_{10}$	$M+H^+$	540,1864	540,1861	-0,6
S. olivaceus	YEPD+SAP	5 coincidences in the DNP	$C_{25}H_{26}O_{10}$	$M+H^+$	487,1599	487,1596	-0,6
S. olivaceus	YEPD+SAP	Jadomycin Ala	$C_{27}H_{25}NO_9$	$M+H^+$	508,1602	508,1595	-1,4
S. olivaceus	YEPD+SAP	Coproporphirin III	$C_{36}H_{38}N_4O_8$	$M+H^+$	655,2762	655,2757	-0,8
S. olivaceus	YEPD+SAP	9-C-D-Olivosyltetrangulol	$C_{25}H_{22}O_7$	$M+H^+$	435,1438	435,1430	-1,8
S. rochei	YEPD+SAP	Fungichromin	$C_{35}H_{58}O_{12}$	$M-H_2O+H^+$	653,3895	653,3891	-0,6
S. rochei	YEPD+SAP	Fungichromin B	$C_{36}H_{60}O_{12}$	$M+H^+$	667,4052	667,4050	-0,3
S. rochei	YEPD+SAP	-	$C_{36}H_{58}O_{13}$	$M-H_2O+H^+$	681,3845	681,3841	-0,6
S. rochei	YEPD+SAP	Actinomycin G4	$C_{61}H_{84}N_{12}O_{17}$	$M+H^+$	629,3111	629,3133	3,5
S. rochei	YEPD+SAP	Actinomycin Xoβ / Actinomycin Xoδ	$C_{62}H_{86}N_{12}O_{17}$	$M+H^+$	636,3180	636,3197	2,7
S. rochei	YEPD+SAP	Actinomycin D	$C_{62}H_{86}N_{12}O_{16}$	$M+H^+$	628,3215	628,3222	1,1
Streptomyces CA-128791	YEPD+SAP	-	$C_8H_6N_2O_3$	$M+H^+$	179,0451	179,0451	0,0
Streptomyces CA-128791	YEPD+SAP	-	$C_{31}H_{37}NO_8$	$M-H_2O+H^+$	534,2486	534,2482	-0,7

Table S2: Characteristics of the *Streptomyces* strains from our collection

Strain	PKS-I	PKS-II	NRPS	Closest Match (Ezbiocloud)*	Similarity*
CA-126369	no	**yes**	no	*Streptomyces glauciniger* NBRC 100913(T)	99,278
CA-126414	**yes**	**yes**	**yes**	*Streptomyces globisporus* subsp. *globisporus* NRRL B-2293(T)	99,587
CA-126521	no	**yes**	**yes**	*Streptomyces caelestis* NRRL 2418(T)	99,383
CA-126542	no	**yes**	**yes**	*Streptomyces misionensis* NBRC 13063(T)	98,759
CA-128791	no	**yes**	no	*Streptomyces pactum* NBRC 13433(T)	100
CA-128875	no	**yes**	**yes**	*Streptomyces halstedii* NBRC 12783(T)	100
CA-128878	**yes**	**yes**	no	*Streptomyces lienomycini* LMG 20091(T)	100
CA-128883	no	**yes**	no	*Streptomyces flavovirens* NBRC 3716(T)	100
CA-128888	no	**yes**	**yes**	*Streptomyces violascens* NBRC 12920(T)	100
CA-128911	no	**yes**	**yes**	*Streptomyces mutomycini* NBRC 100999(T)	99,897
CA-129322	**yes**	**yes**	**yes**	*Streptomyces gelaticus* NRRL B-2928(T)	97,708
CA-131129	no	**yes**	**yes**	*Streptomyces tendae* ATCC 19812(T)	99,879

*16S RNA sequencing
https://www.ezbiocloud.net/identify

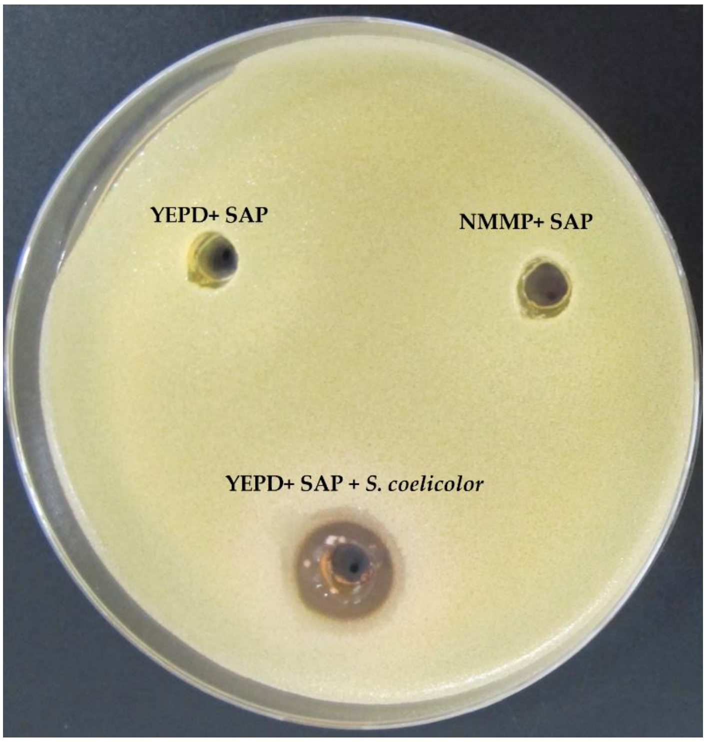

Figure S1: Antibiotic activity against *M. lute*us. In each well 100 μL of the indicated media + xylem sap (SAP) has been added. As a positive control 100 μL of a culture of *S. coelicolor* in YEPD + SAP was used.

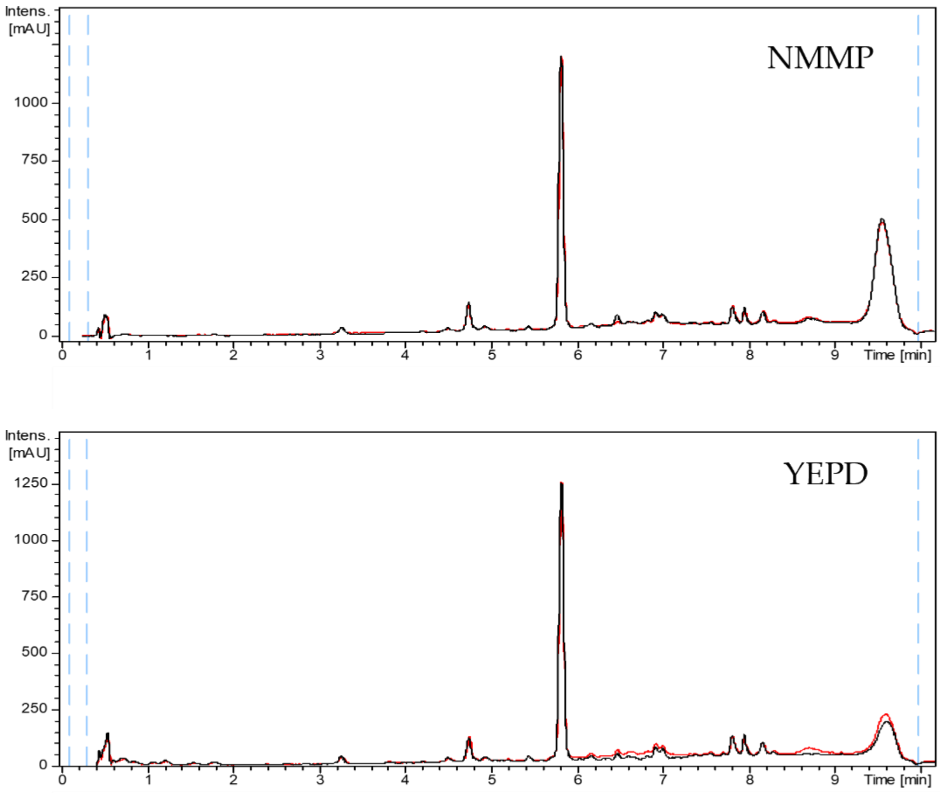

Figure S2: Metabolites present in culture media, NMMP or YEPD diluted ½ with xylem sap (red) or with water (black) detected by UV-Vis absorbance (200-900 nm).

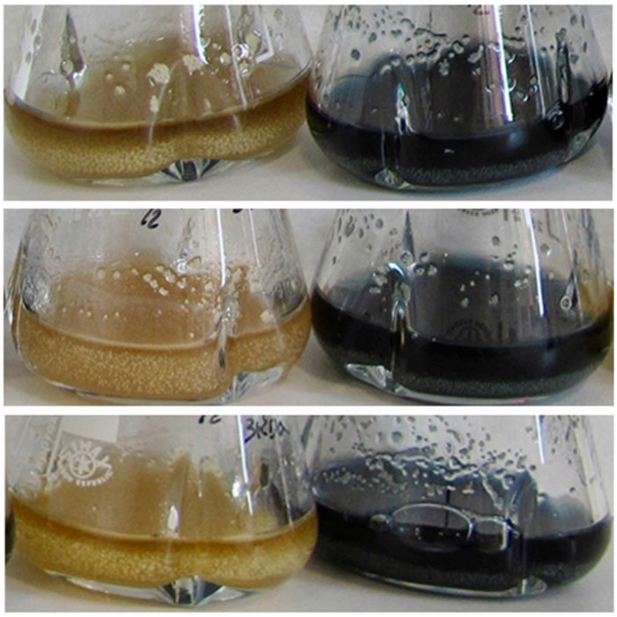

Figure S3: ACT production (blue color) in NMMP medium by *S. coelicolor* induced by SAP (upper right). Effect soluble and insoluble fractions of SAP in methanol (middle) over the production of ACT. Effect of the retained fraction (R3) or eluted fraction (E3) (MWCO 3 kDa) from the methanol insoluble fraction on ACT production.

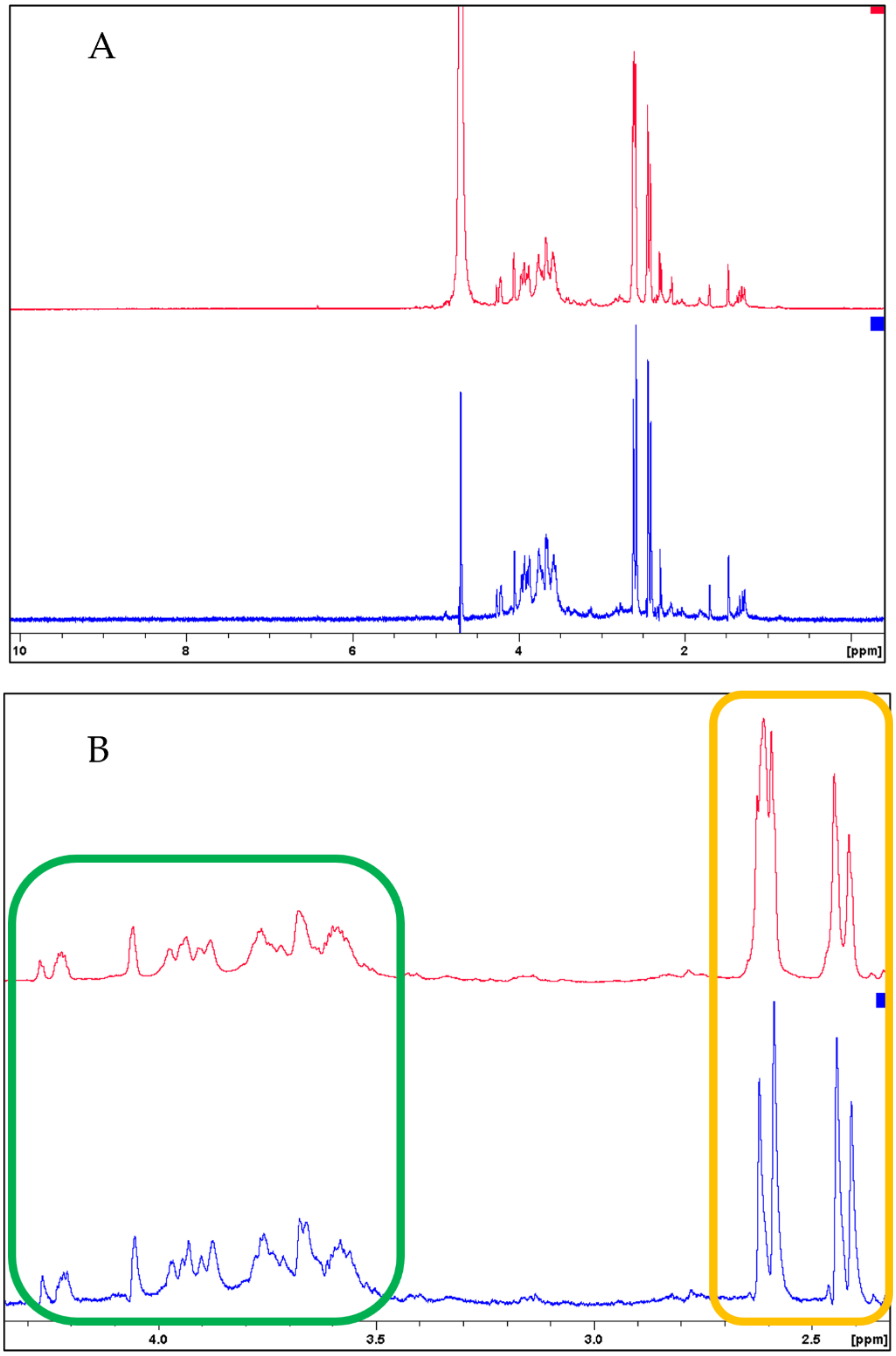

Figure S4: **A)** Standard ^{1}H NMR (red trace) and diffusion filtered ^{1}H NMR (blue trace) spectra of the active fraction (fraction 6) from the grapevine xylem sap LH-20 fractionation (registered in D_2O, 24 °C at 500 MHz) **B)** Expanded regions of the standard ^{1}H NMR (red trace) and diffusion filtered ^{1}H NMR (blue trace) spectra highlighting the signals associated to citrate (orange box) and di-D-fructose dianhydrides (green box). The citrate signals perfectly matched those reported for citric acid in the Biological Magnetic Resonance Databank (*doi:10.13018/BMSE000076*).

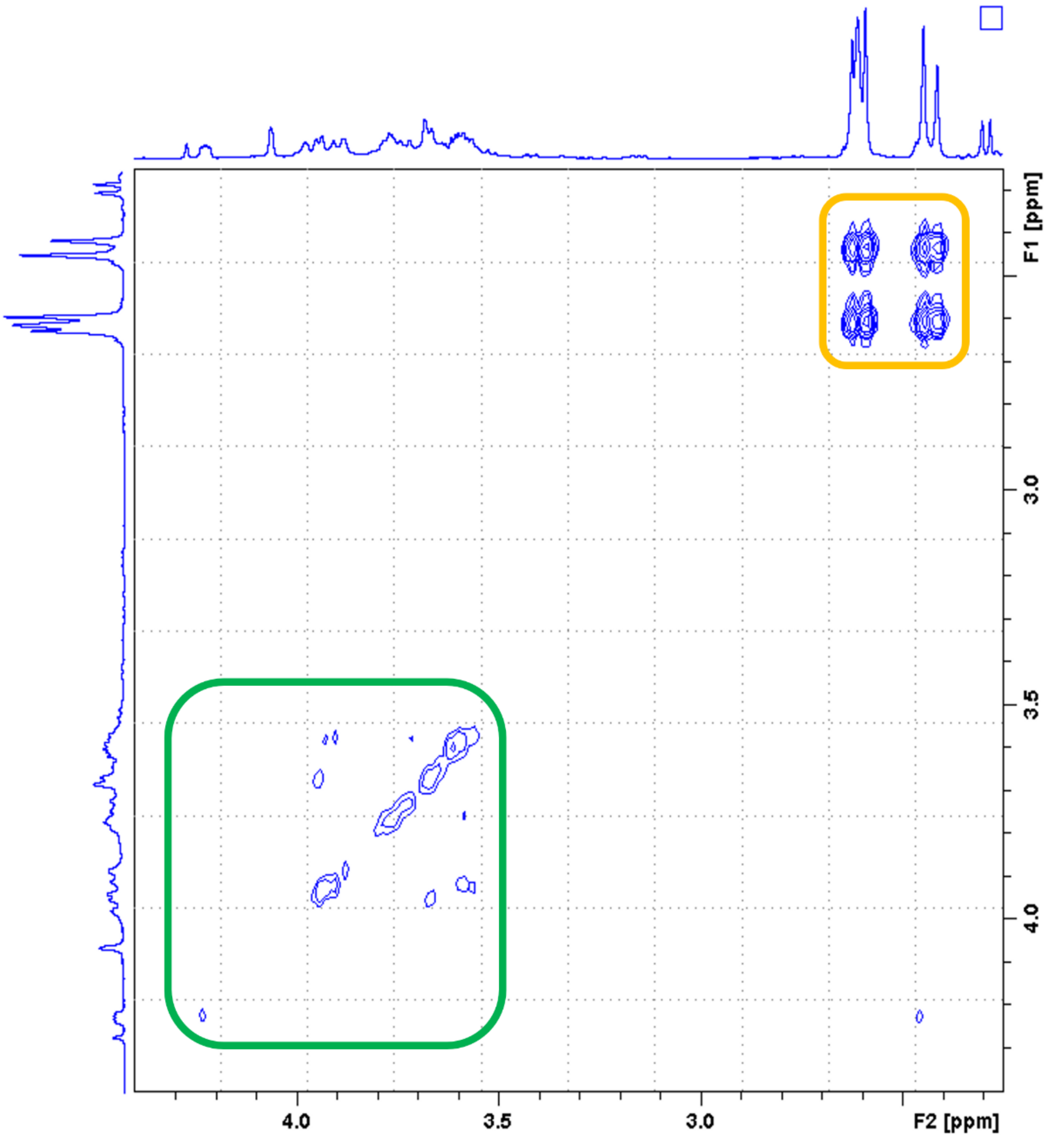

Figure S5: Zoomed region of the COSY spectrum of the active fraction (fraction 6) highlighting the signals associated to citrate (orange box) and di-D-fructose dianhydrides (green box). The citrate signals perfectly matched those reported for citric acid in the Biological Magnetic Resonance Databank (*doi:10.13018/BMSE000076*).

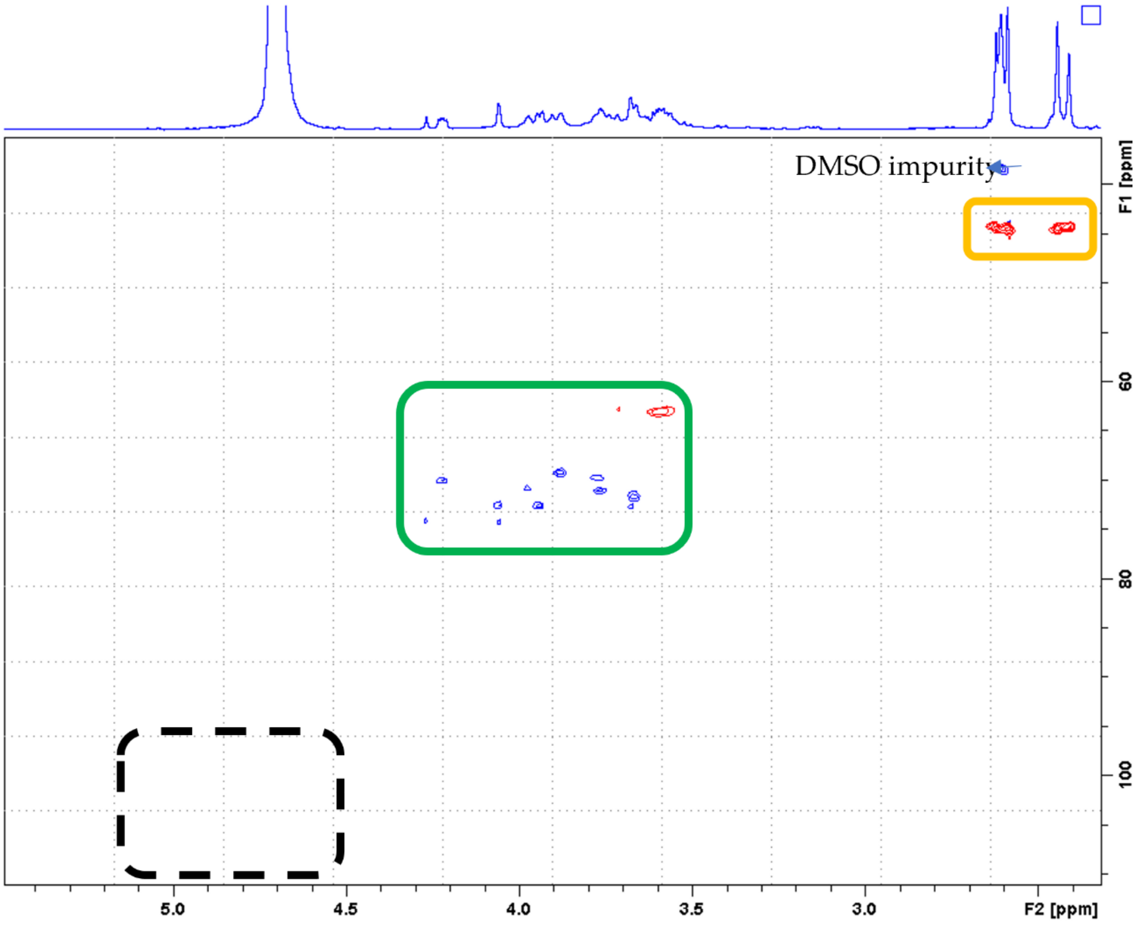

Figure S6: Edited HSQC spectrum highlighting the signals of the active fraction (fraction 6) associated to citrate (orange box) and di-D-fructose dianhydrides (green box). Clearly no anomeric methine signal is observed on its typical region (dashed black box). The citrate signals perfectly matched those reported for citric acid in the Biological Magnetic Resonance Databank (*doi:10.13018/BMSE000076*).

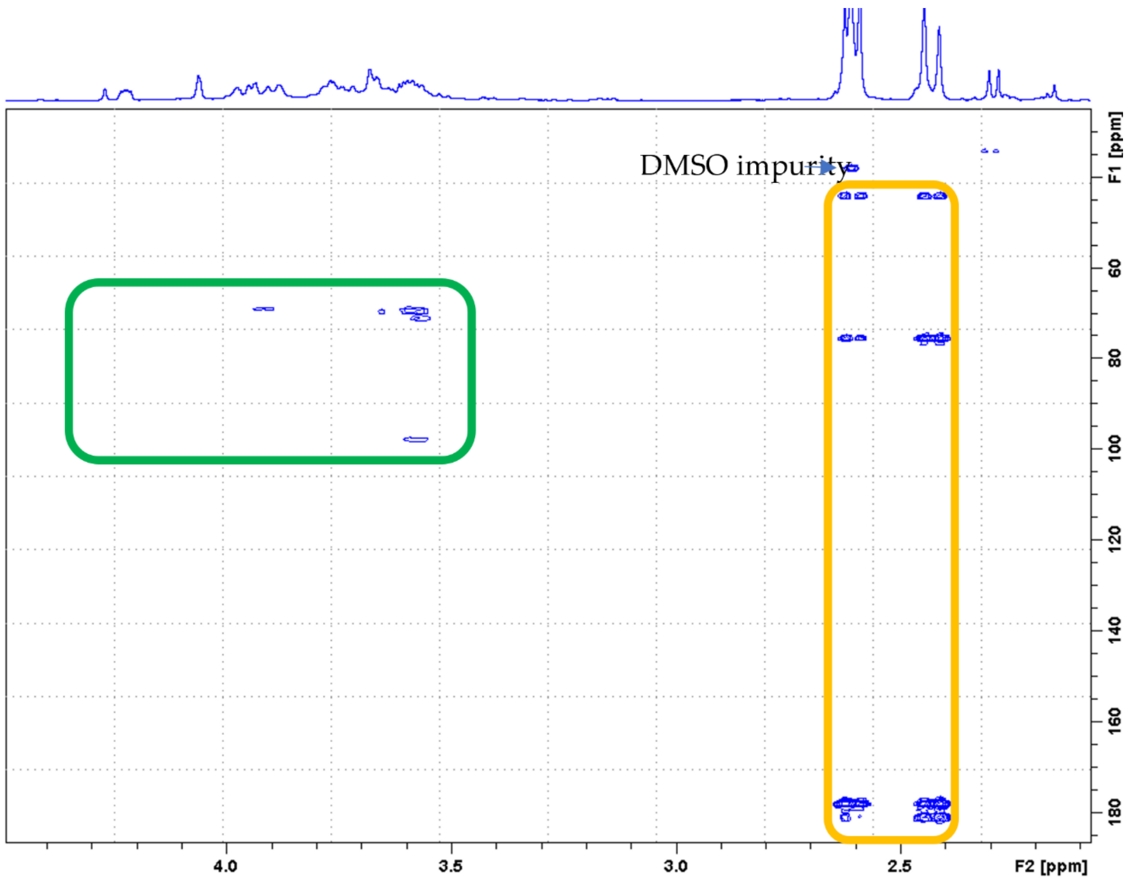

Figure S7: Zoomed región of the HMBC spectrum of the active fraction (fraction 6) highlighting the signals associated to citrate (orange box) and di-D-fructose dianhydrides (green box). The citrate is much more concentrated than the carbohydrate and thus renders very strong long-range correlation cross peaks. The citrate signals perfectly matched those reported for citric acid in the Biological Magnetic Resonance Databank (*doi:10.13018/BMSE000076*).

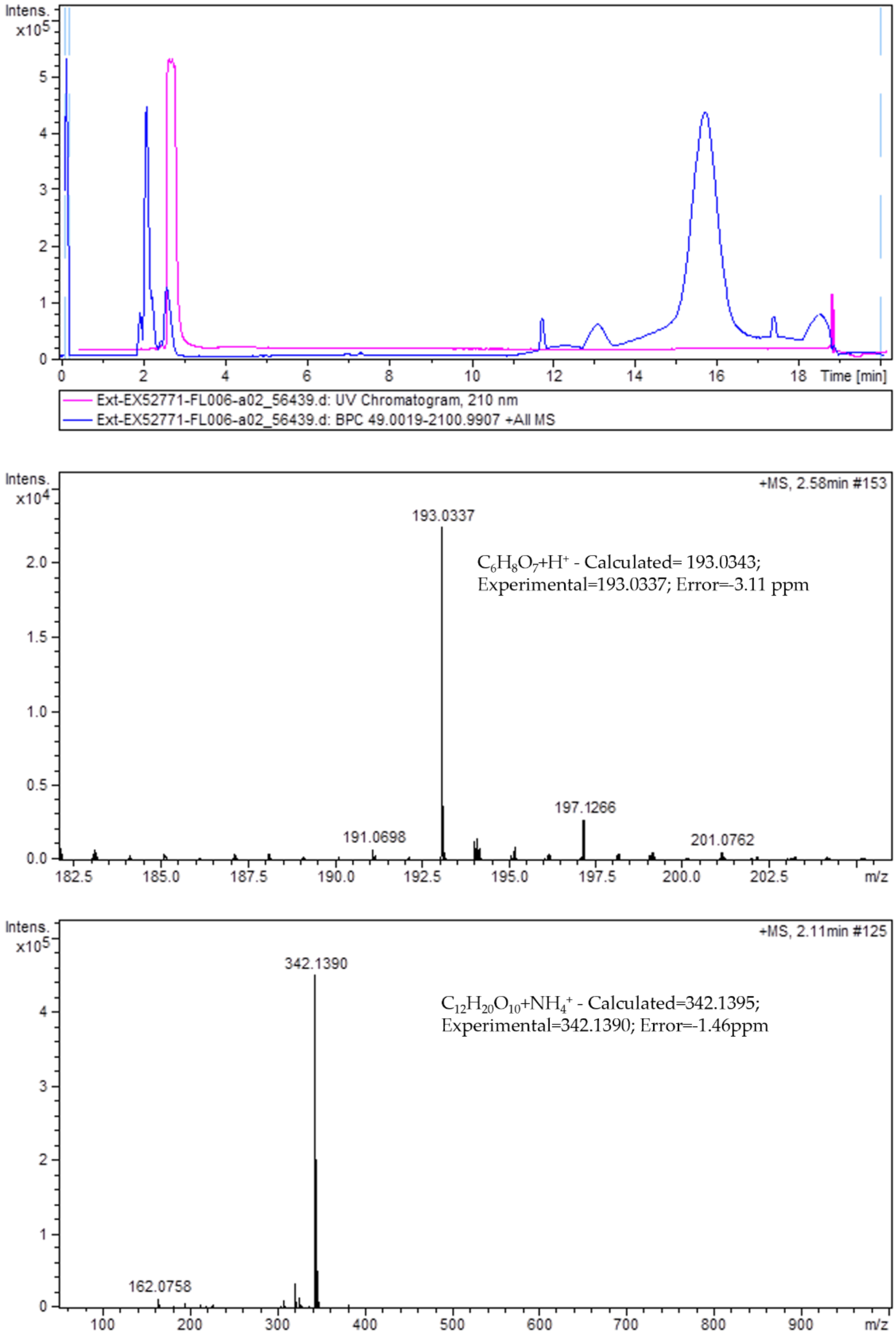

Figure S8: LC-HRMS analysis of the active fraction (fraction 6) from the grapevine xylem sap LH-20 fractionation. The corresponding MS spectra of citric acid ($C_6H_8O_7$) and the di-D-fructose dianhydrides ($C_{12}H_{20}O_{10}$) are shown. Although citrate is present at much higher concentration in the sample (as revealed by NMR), its response in positive ionization mode is poorer than the carbohydrate due to its triacidic nature.

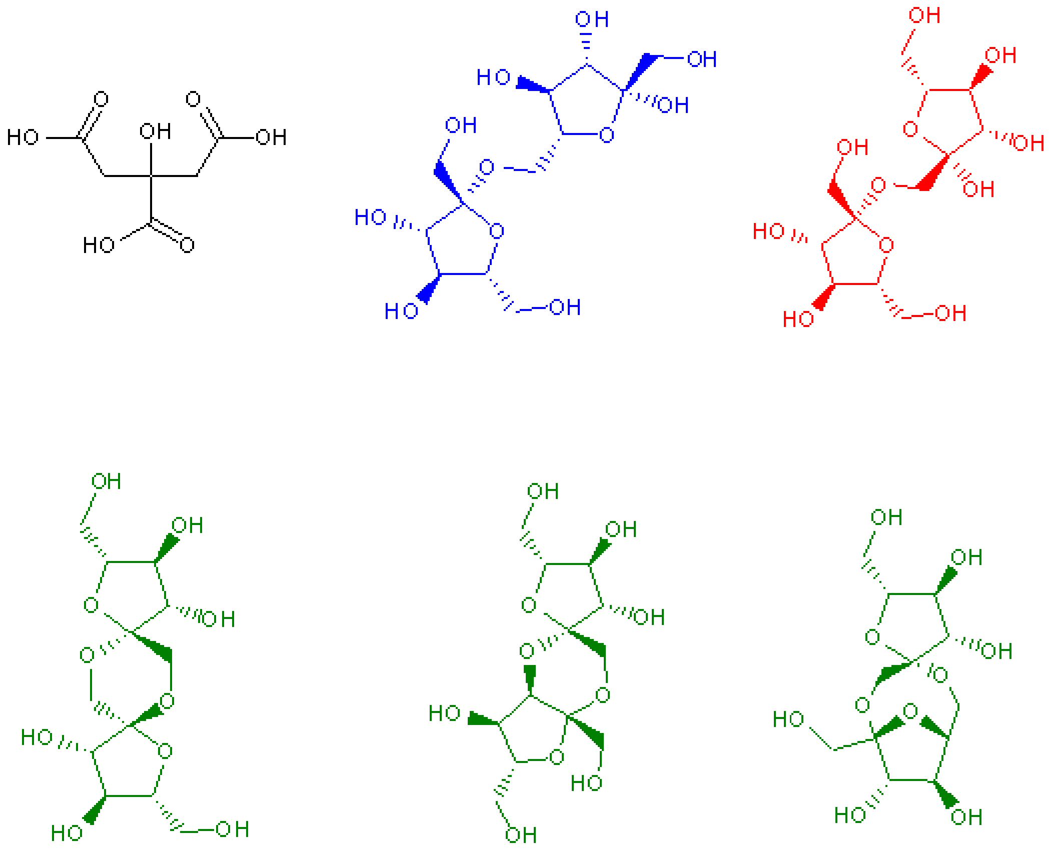

Figure S9: Chemical structure of citric acid (black), levanbiose (blue), inulobiose (red) and some representative di-D-fructose dianhydrides (green) reported to be formed during thermal treatment of the fructan inulin in the presence of citric acid (Christian et al. *J. Agric. Food Chem*. 2000, 48, 5, 1823–1837; https://doi.org/10.1021/jf9911186).

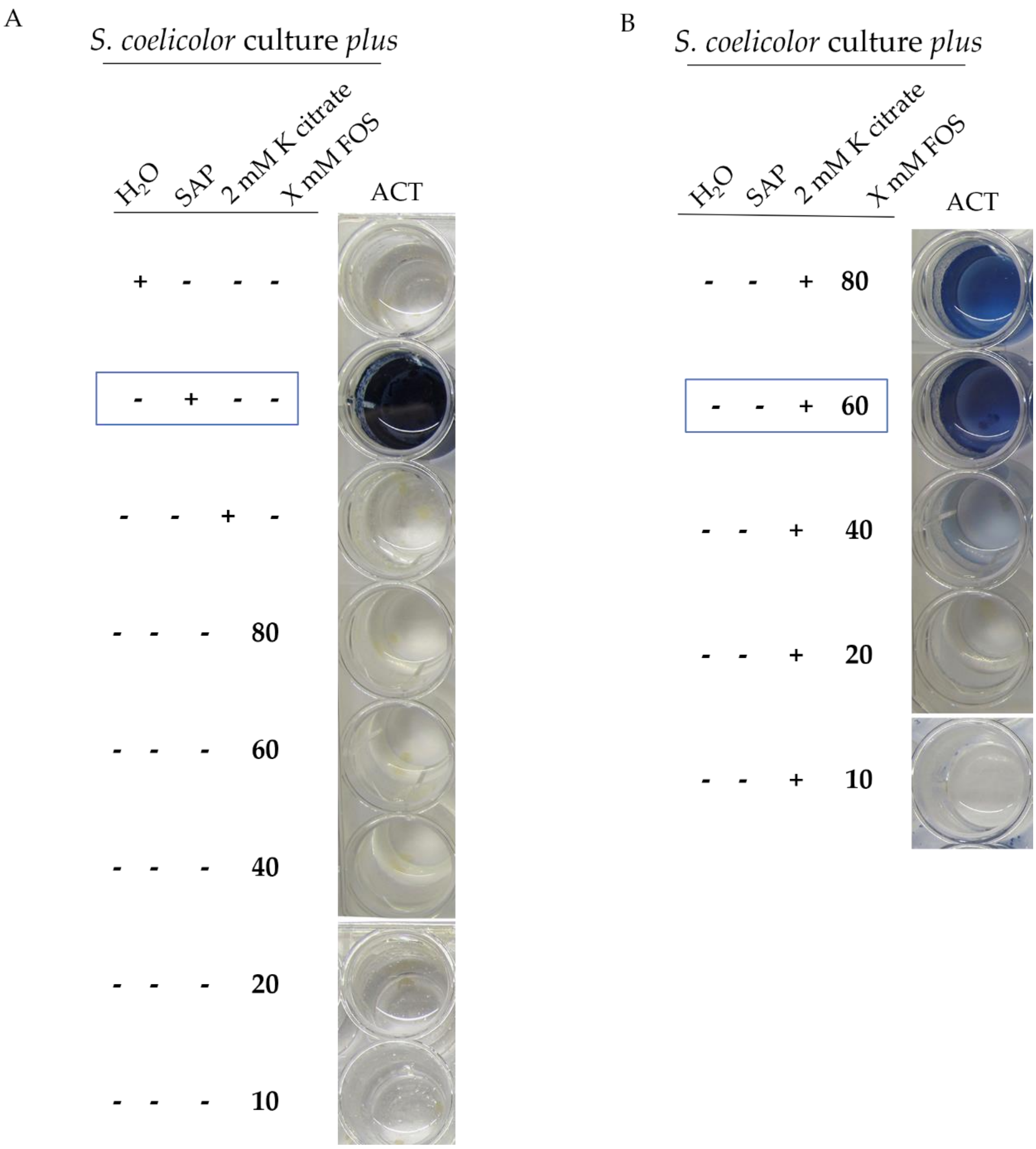

Figure S10: (A) ACT production (blue color) by *S. coelicolor* induced by xylem sap (SAP), potassium citrate (K citrate) or different concentrations of fructooligosaccharides (FOS) in NMMP medium. (B) ACT production by *S. coelicolor* induced by 2mM potassium citrate (K citrate) and different concentrations of fructooligosaccharides (FOS).
